# Supplementary material for: Study of the Genetic Expression of Antiretroviral Restriction Factors and Acute Phase Proteins in Cattle Infected with Bovine Leukemia Virus
Source: Pathogens. 2023 Mar 29;12(4):529. doi: 10.3390/pathogens12040529 (PMC10146972; doi:10.3390/pathogens12040529)
Supplement: Supplementary file 1 [file pathogens-12-00529-s001.zip › Figure S2.pdf]

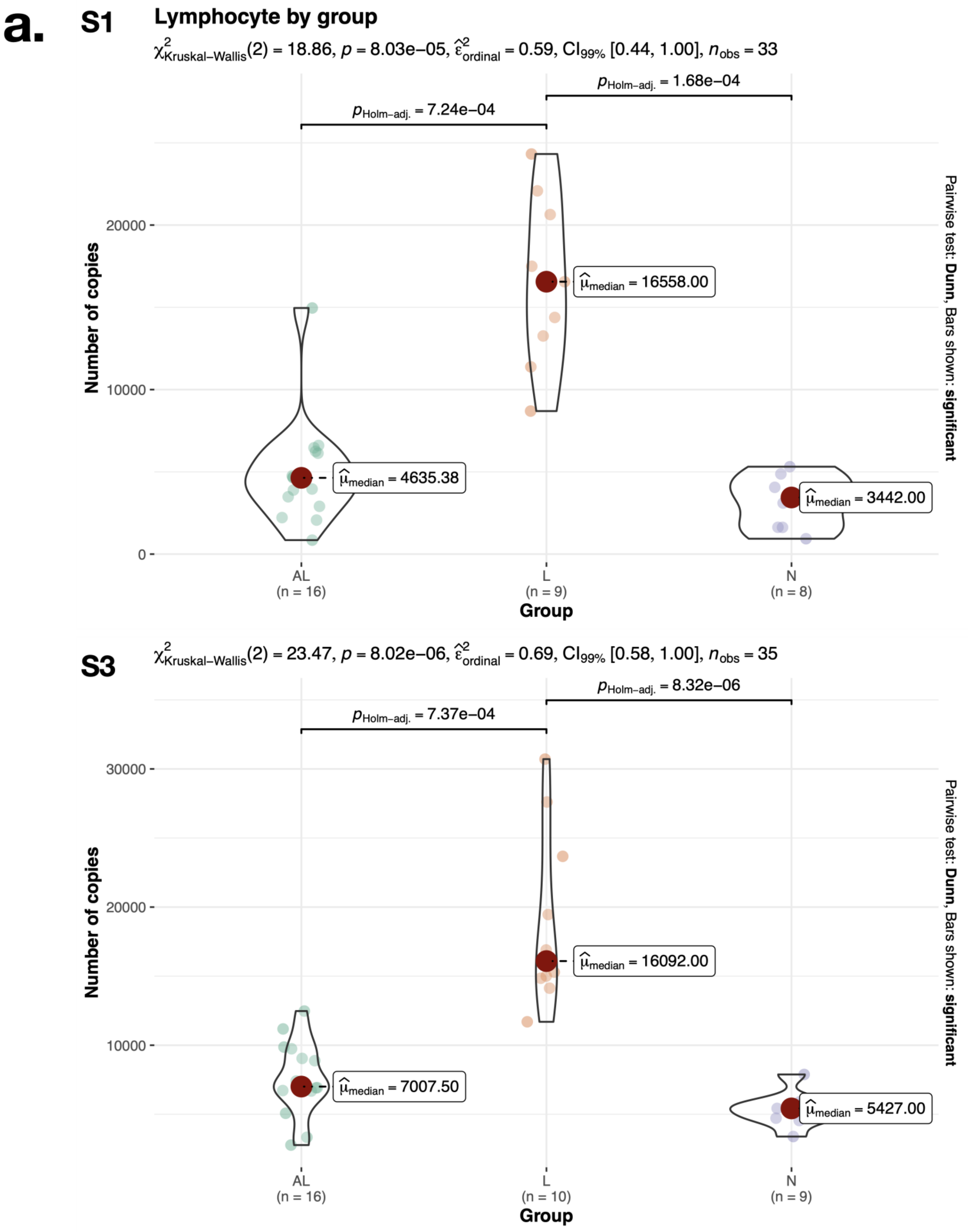
a.
S1
Lymphocyte by group
$\chi^2_{Kruskal-Wallis}(2) = 18.86$, $p = 8.03e-05$, $\hat{\varepsilon}^2_{ordinal} = 0.59$, $CI_{99\%}$ [0.44, 1.00], $n_{obs} = 33$
$p_{Holm-adj.} = 1.68e-04$
$p_{Holm-adj.} = 7.24e-04$
$\hat{\mu}_{median} = 16558.00$
$\hat{\mu}_{median} = 4635.38$
$\hat{\mu}_{median} = 3442.00$
Number of copies
20000
10000
0
AL
(n = 16)
L
(n = 9)
N
(n = 8)
Group
Pairwise test: **Dunn**, Bars shown: **significant**
S3
$\chi^2_{Kruskal-Wallis}(2) = 23.47$, $p = 8.02e-06$, $\hat{\varepsilon}^2_{ordinal} = 0.69$, $CI_{99\%}$ [0.58, 1.00], $n_{obs} = 35$
$p_{Holm-adj.} = 8.32e-06$
$p_{Holm-adj.} = 7.37e-04$
$\hat{\mu}_{median} = 16092.00$
$\hat{\mu}_{median} = 7007.50$
$\hat{\mu}_{median} = 5427.00$
Number of copies
30000
20000
10000
AL
(n = 16)
L
(n = 10)
N
(n = 9)
Group
Pairwise test: **Dunn**, Bars shown: **significant**

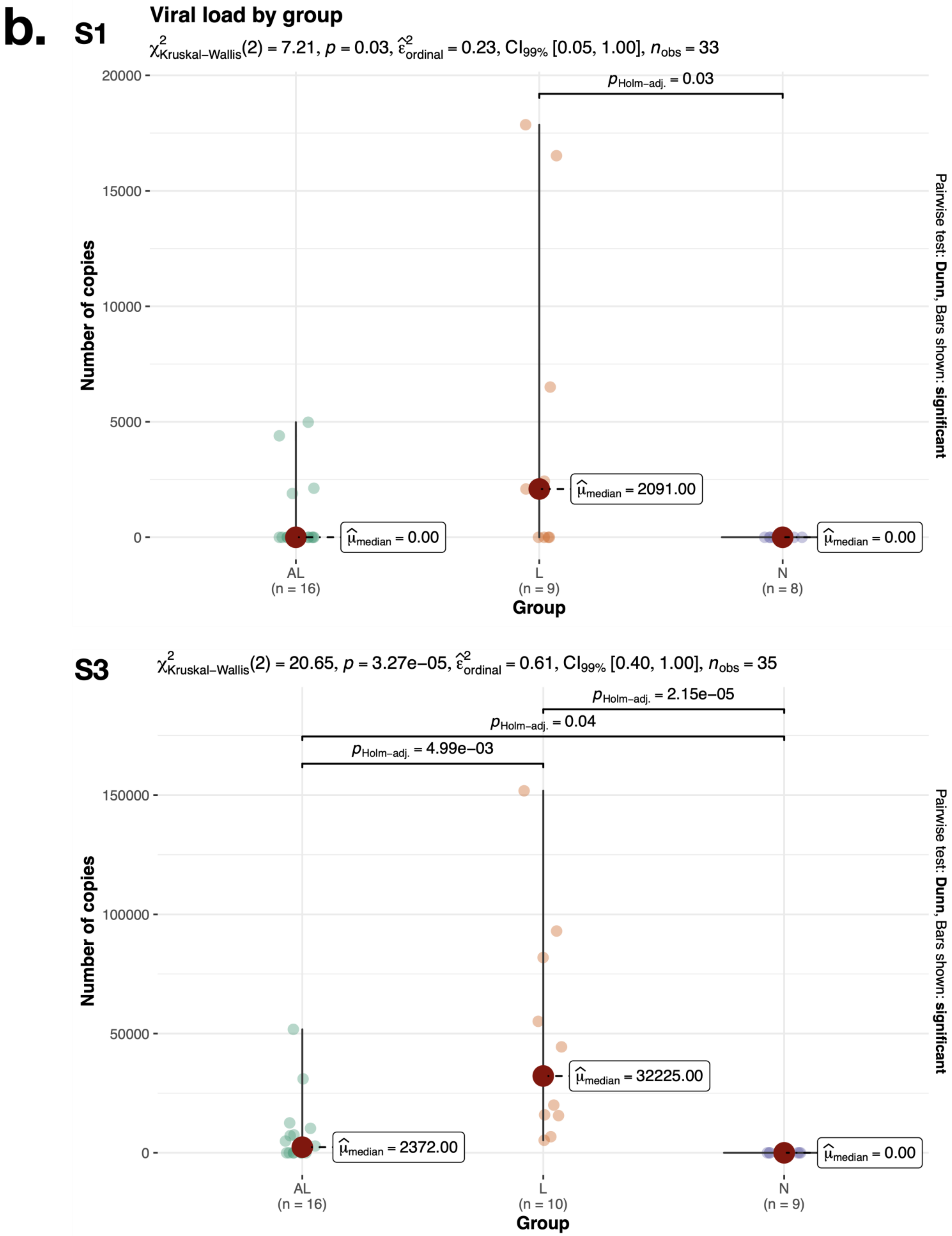
b.
S1
Viral load by group
$\chi^2_{\text{Kruskal-Wallis}}(2) = 7.21$, $p = 0.03$, $\widehat{\varepsilon}^2_{\text{ordinal}} = 0.23$, $\text{CI}_{99\%}$ [0.05, 1.00], $n_{\text{obs}} = 33$
$p_{\text{Holm-adj.}} = 0.03$
Number of copies
20000
15000
10000
5000
0
$\widehat{\mu}_{\text{median}} = 0.00$
$\widehat{\mu}_{\text{median}} = 2091.00$
$\widehat{\mu}_{\text{median}} = 0.00$
AL
(n = 16)
L
(n = 9)
N
(n = 8)
Group
Pairwise test: **Dunn**, Bars shown: **significant**
S3
$\chi^2_{\text{Kruskal-Wallis}}(2) = 20.65$, $p = 3.27\text{e}{-05}$, $\widehat{\varepsilon}^2_{\text{ordinal}} = 0.61$, $\text{CI}_{99\%}$ [0.40, 1.00], $n_{\text{obs}} = 35$
$p_{\text{Holm-adj.}} = 2.15\text{e}{-05}$
$p_{\text{Holm-adj.}} = 0.04$
$p_{\text{Holm-adj.}} = 4.99\text{e}{-03}$
Number of copies
150000
100000
50000
0
$\widehat{\mu}_{\text{median}} = 2372.00$
$\widehat{\mu}_{\text{median}} = 32225.00$
$\widehat{\mu}_{\text{median}} = 0.00$
AL
(n = 16)
L
(n = 10)
N
(n = 9)
Group
Pairwise test: **Dunn**, Bars shown: **significant**

c. **S1**

Proviral load by group

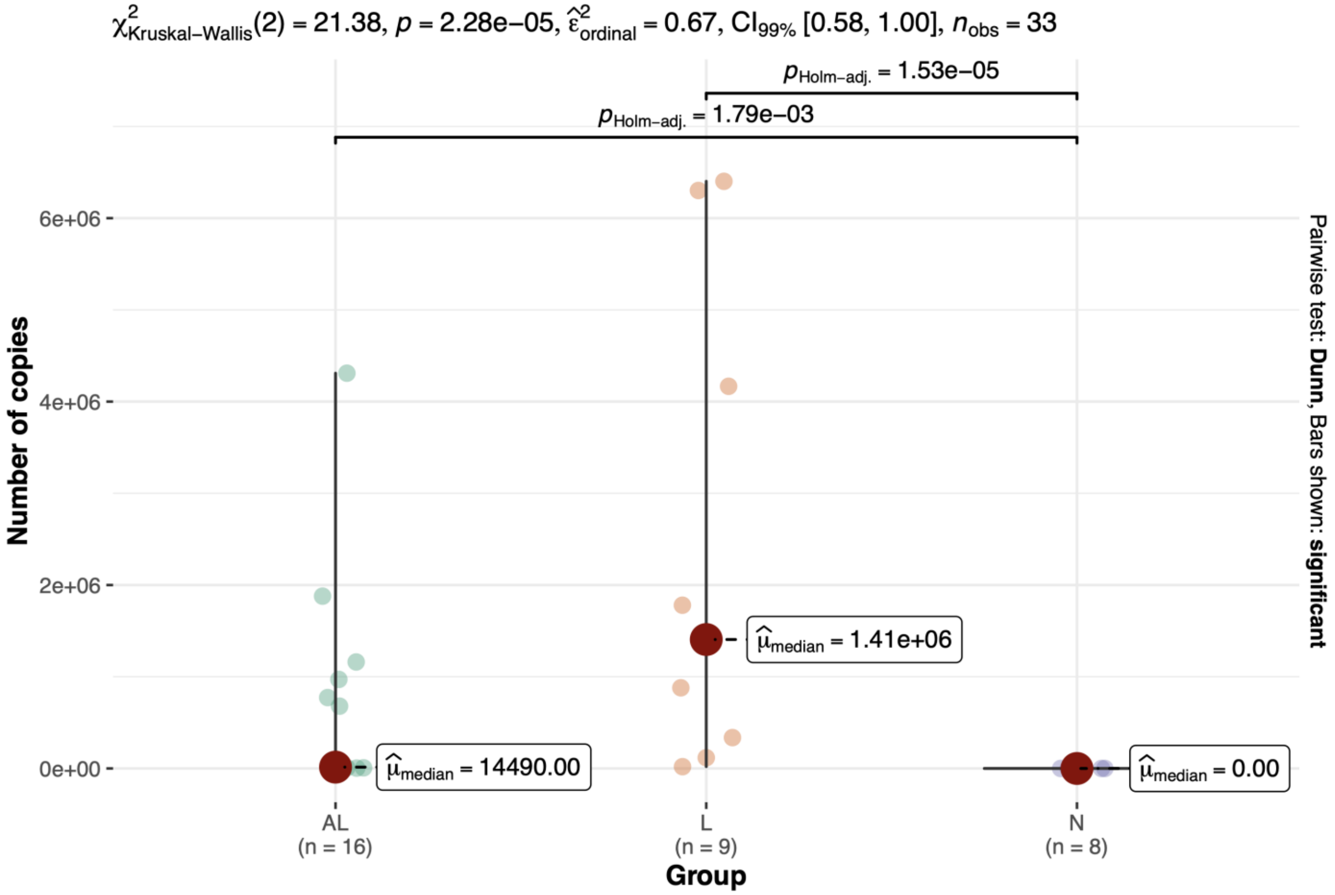

S3

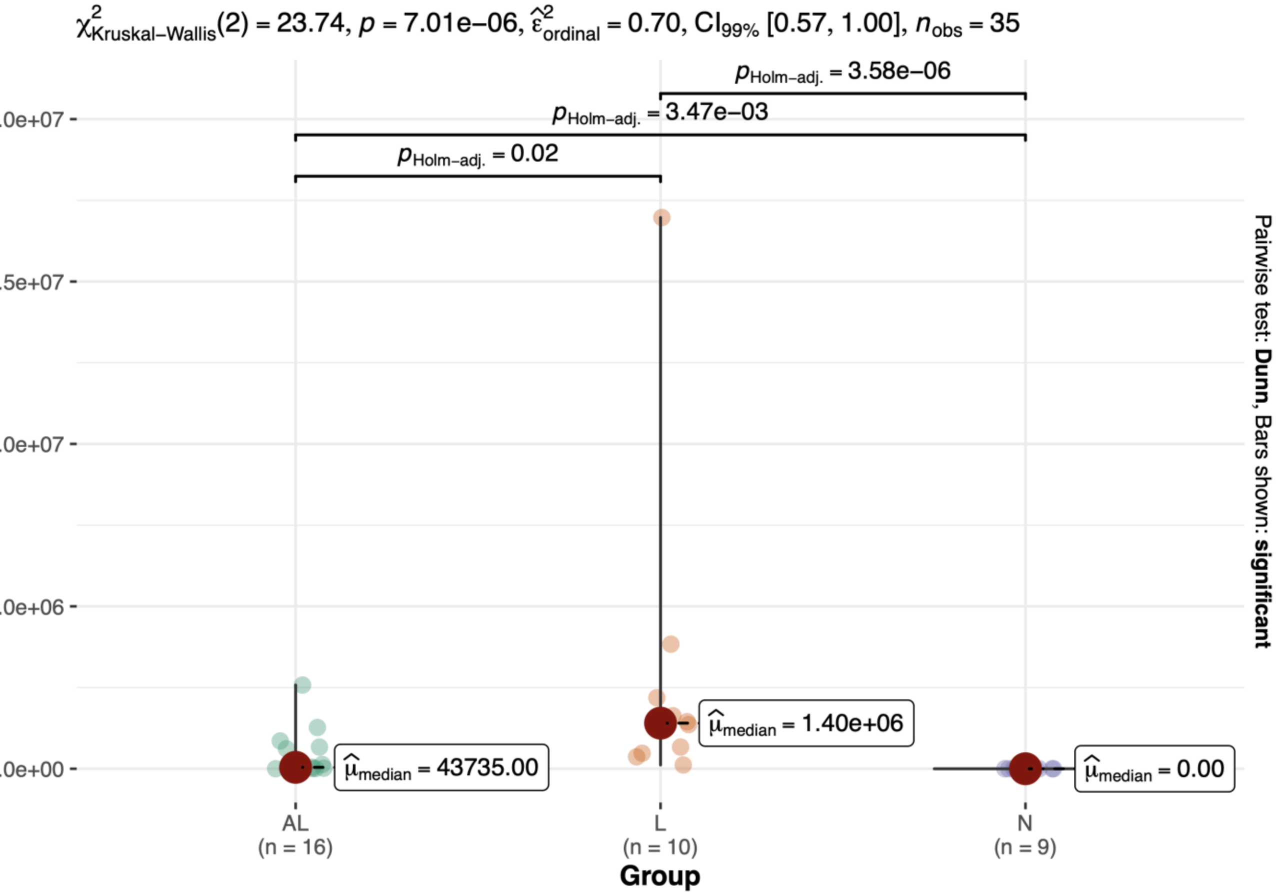

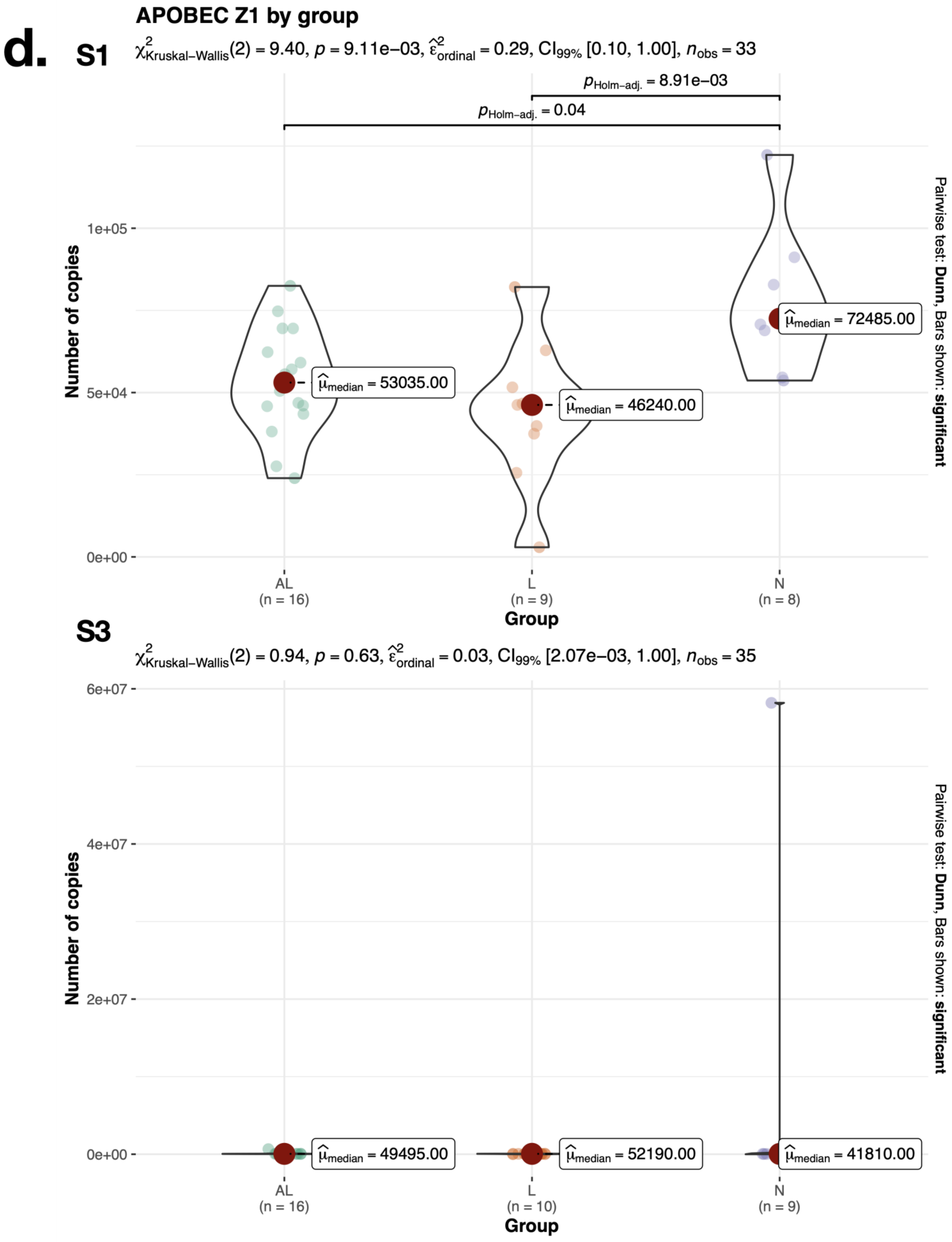
d.
APOBEC Z1 by group
S1
χ²Kruskal–Wallis(2) = 9.40, p = 9.11e−03, ε̂²ordinal = 0.29, CI99% [0.10, 1.00], nobs = 33
pHolm−adj. = 8.91e−03
pHolm−adj. = 0.04
μ̂median = 53035.00
μ̂median = 46240.00
μ̂median = 72485.00
Number of copies
1e+05
5e+04
0e+00
AL
(n = 16)
L
(n = 9)
N
(n = 8)
Group
Pairwise test: Dunn, Bars shown: significant
S3
χ²Kruskal–Wallis(2) = 0.94, p = 0.63, ε̂²ordinal = 0.03, CI99% [2.07e−03, 1.00], nobs = 35
6e+07
4e+07
2e+07
0e+00
μ̂median = 49495.00
μ̂median = 52190.00
μ̂median = 41810.00
AL
(n = 16)
L
(n = 10)
N
(n = 9)
Group
Pairwise test: Dunn, Bars shown: significant

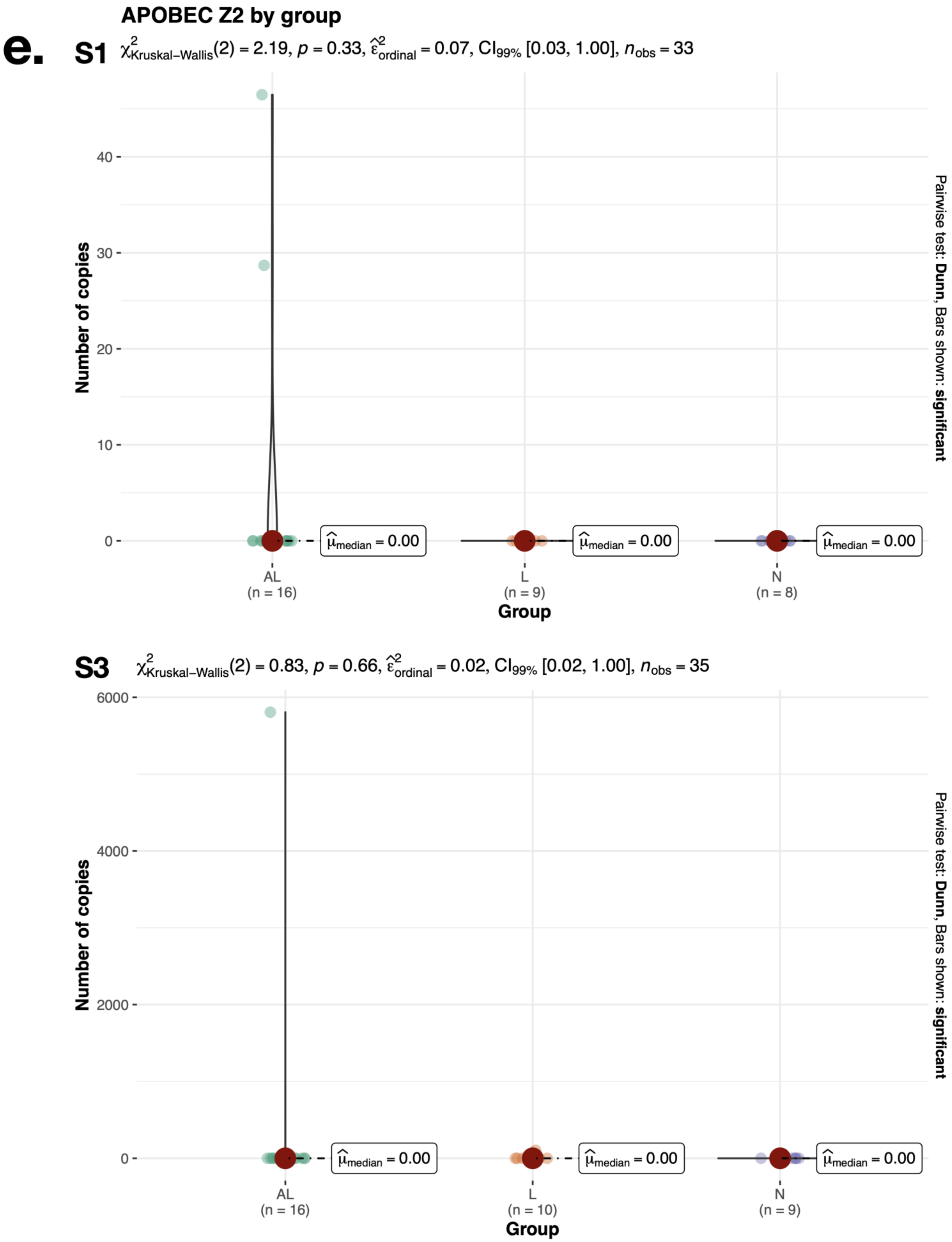

e.
APOBEC Z2 by group
S1
$\chi^2_{\text{Kruskal-Wallis}}(2) = 2.19$, $p = 0.33$, $\hat{\varepsilon}^2_{\text{ordinal}} = 0.07$, $\text{CI}_{99\%}$ [0.03, 1.00], $n_{\text{obs}} = 33$
Number of copies
Pairwise test: Dunn, Bars shown: significant
$\hat{\mu}_{\text{median}} = 0.00$
$\hat{\mu}_{\text{median}} = 0.00$
$\hat{\mu}_{\text{median}} = 0.00$
AL
(n = 16)
L
(n = 9)
N
(n = 8)
Group
S3
$\chi^2_{\text{Kruskal-Wallis}}(2) = 0.83$, $p = 0.66$, $\hat{\varepsilon}^2_{\text{ordinal}} = 0.02$, $\text{CI}_{99\%}$ [0.02, 1.00], $n_{\text{obs}} = 35$
Number of copies
Pairwise test: Dunn, Bars shown: significant
$\hat{\mu}_{\text{median}} = 0.00$
$\hat{\mu}_{\text{median}} = 0.00$
$\hat{\mu}_{\text{median}} = 0.00$
AL
(n = 16)
L
(n = 10)
N
(n = 9)
Group

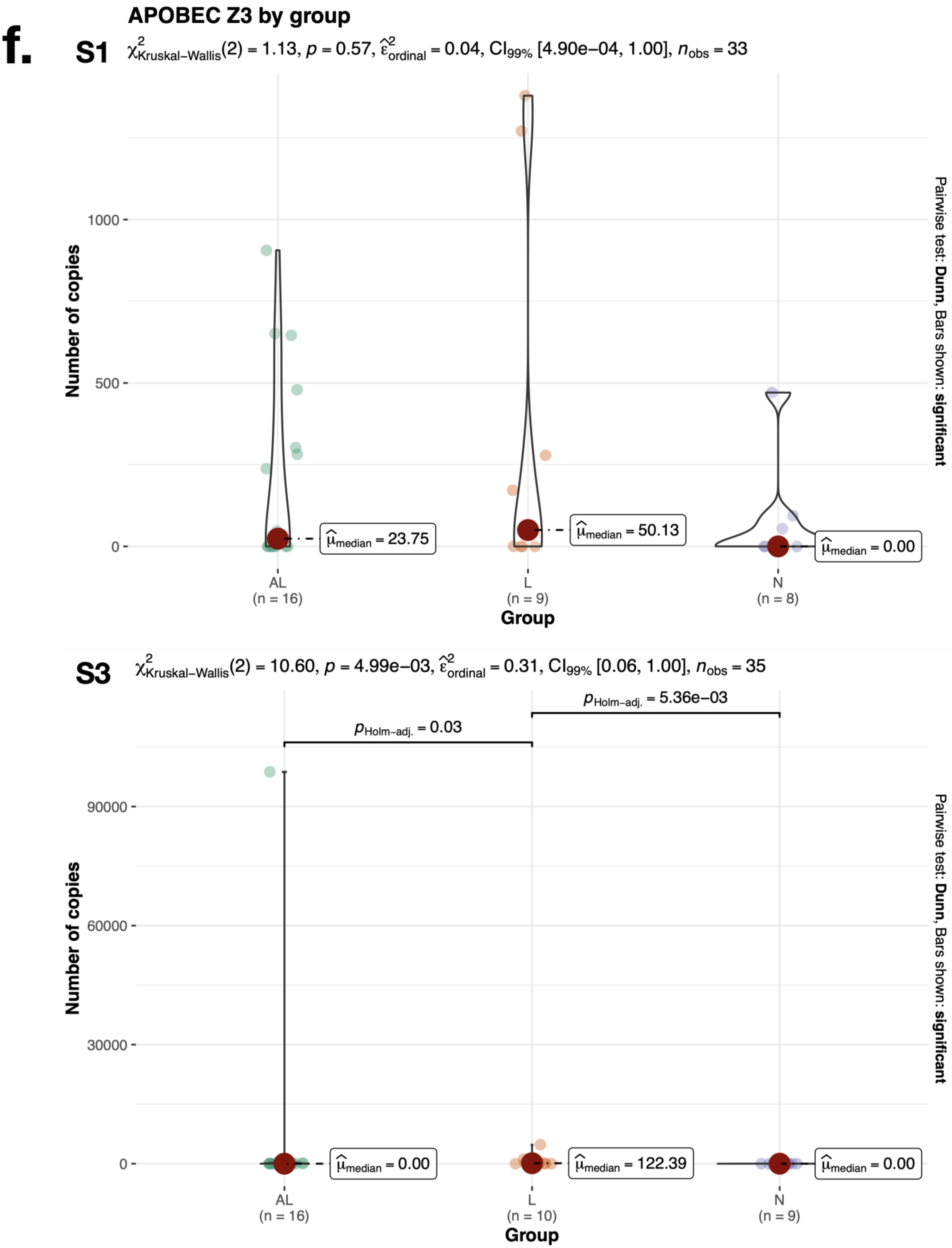

f.
APOBEC Z3 by group
S1
χ²Kruskal–Wallis(2) = 1.13, p = 0.57, ε̂²ordinal = 0.04, CI99% [4.90e–04, 1.00], nobs = 33
Number of copies
1000
500
0
μ̂median = 23.75
μ̂median = 50.13
μ̂median = 0.00
AL
(n = 16)
L
(n = 9)
N
(n = 8)
Group
Pairwise test: Dunn, Bars shown: significant
S3
χ²Kruskal–Wallis(2) = 10.60, p = 4.99e–03, ε̂²ordinal = 0.31, CI99% [0.06, 1.00], nobs = 35
pHolm–adj. = 5.36e–03
pHolm–adj. = 0.03
Number of copies
90000
60000
30000
0
μ̂median = 0.00
μ̂median = 122.39
μ̂median = 0.00
AL
(n = 16)
L
(n = 10)
N
(n = 9)
Group
Pairwise test: Dunn, Bars shown: significant

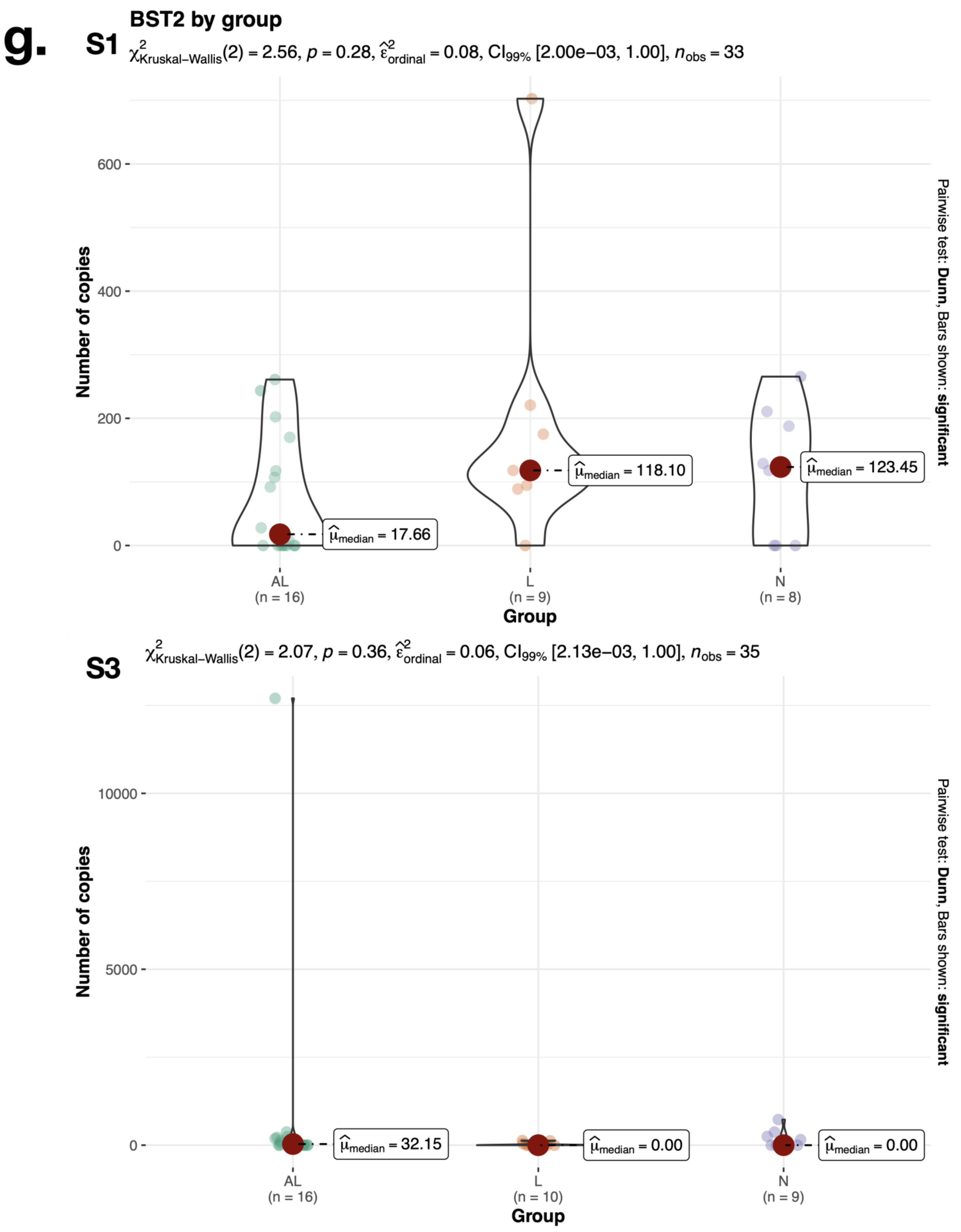
g.
S1
BST2 by group
$\chi^2_{Kruskal-Wallis}(2) = 2.56$, $p = 0.28$, $\hat{\epsilon}^2_{ordinal} = 0.08$, $CI_{99\%}$ [2.00e−03, 1.00], $n_{obs} = 33$
Number of copies
600
400
200
0
$\hat{\mu}_{median} = 17.66$
$\hat{\mu}_{median} = 118.10$
$\hat{\mu}_{median} = 123.45$
AL (n = 16)
L (n = 9)
N (n = 8)
Group
Pairwise test: **Dunn**, Bars shown: **significant**
S3
$\chi^2_{Kruskal-Wallis}(2) = 2.07$, $p = 0.36$, $\hat{\epsilon}^2_{ordinal} = 0.06$, $CI_{99\%}$ [2.13e−03, 1.00], $n_{obs} = 35$
Number of copies
10000
5000
0
$\hat{\mu}_{median} = 32.15$
$\hat{\mu}_{median} = 0.00$
$\hat{\mu}_{median} = 0.00$
AL (n = 16)
L (n = 10)
N (n = 9)
Group
Pairwise test: **Dunn**, Bars shown: **significant**

h.

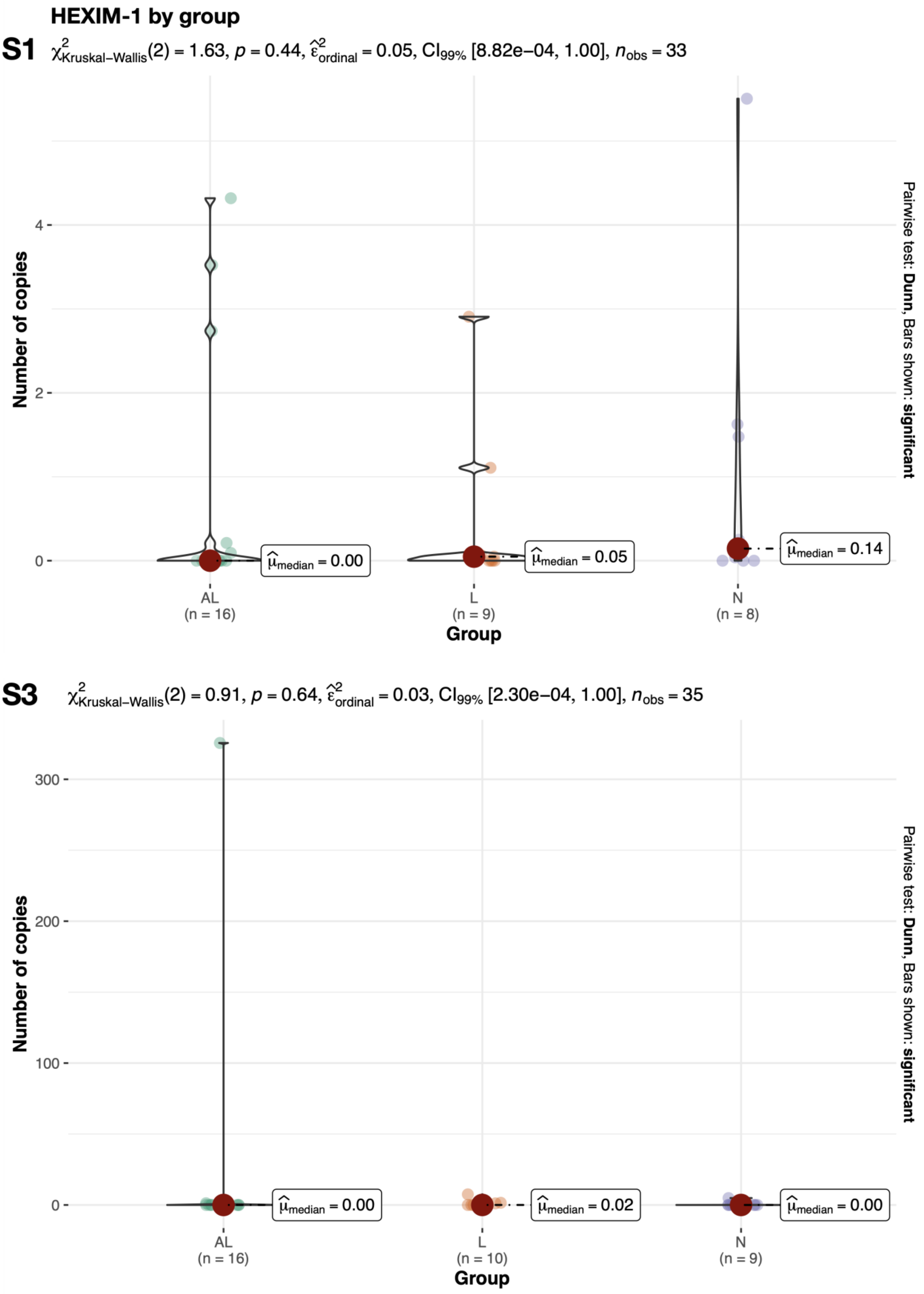

HEXIM-1 by group
S1
$\chi^2_{\text{Kruskal-Wallis}}(2) = 1.63, p = 0.44, \hat{\varepsilon}^2_{\text{ordinal}} = 0.05, \text{CI}_{99\%}$ [8.82e−04, 1.00], $n_{\text{obs}} = 33$
Number of copies
$\hat{\mu}_{\text{median}} = 0.00$
$\hat{\mu}_{\text{median}} = 0.05$
$\hat{\mu}_{\text{median}} = 0.14$
AL
(n = 16)
L
(n = 9)
N
(n = 8)
Group
Pairwise test: **Dunn**, Bars shown: **significant**
S3
$\chi^2_{\text{Kruskal-Wallis}}(2) = 0.91, p = 0.64, \hat{\varepsilon}^2_{\text{ordinal}} = 0.03, \text{CI}_{99\%}$ [2.30e−04, 1.00], $n_{\text{obs}} = 35$
Number of copies
$\hat{\mu}_{\text{median}} = 0.00$
$\hat{\mu}_{\text{median}} = 0.02$
$\hat{\mu}_{\text{median}} = 0.00$
AL
(n = 16)
L
(n = 10)
N
(n = 9)
Group
Pairwise test: **Dunn**, Bars shown: **significant**

i.

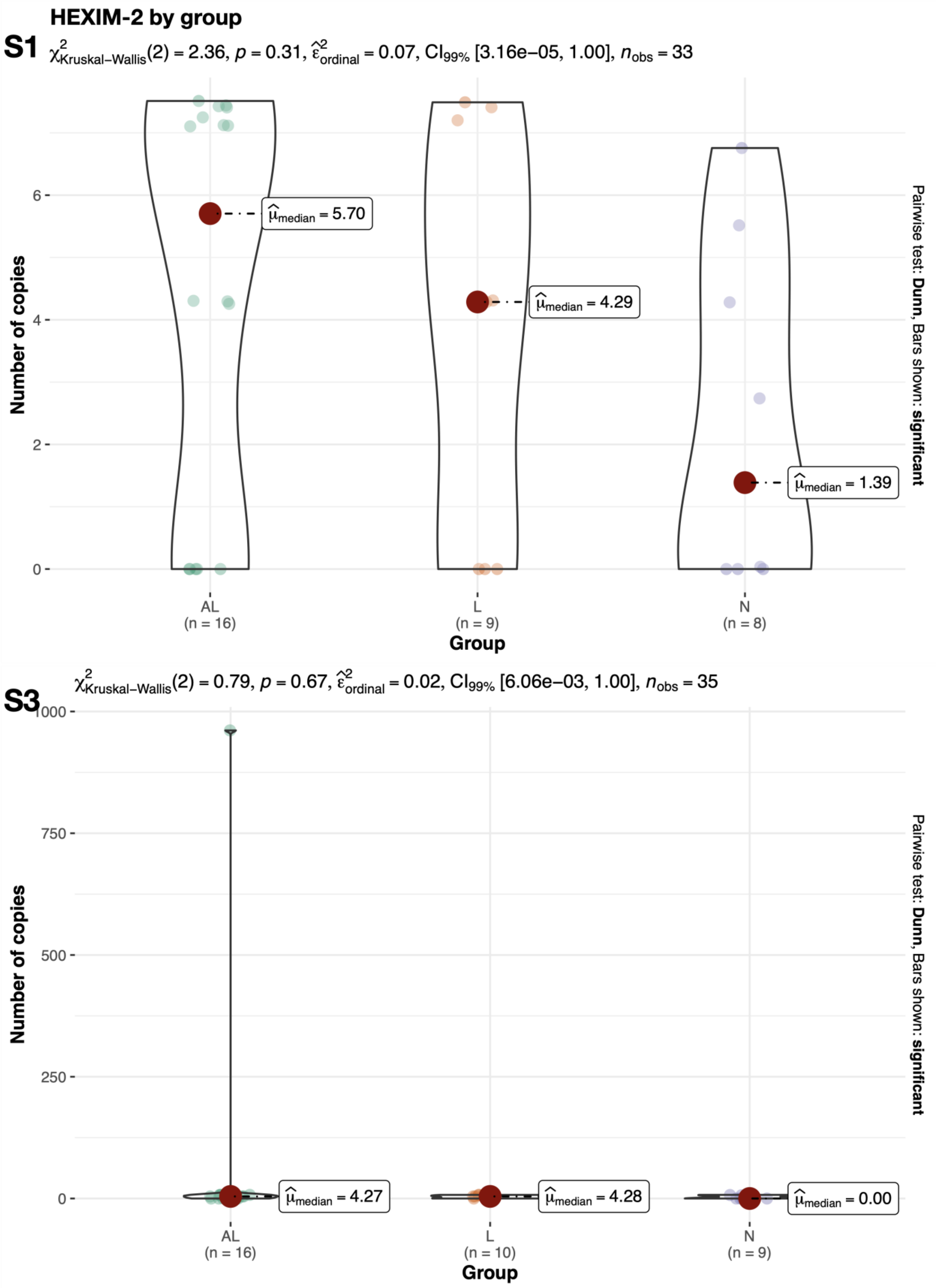
HEXIM-2 by group
S1
$\chi^2_{Kruskal-Wallis}(2) = 2.36$, $p = 0.31$, $\hat{\epsilon}^2_{ordinal} = 0.07$, $CI_{99\%}$ [3.16e−05, 1.00], $n_{obs} = 33$
Number of copies
$\hat{\mu}_{median} = 5.70$
$\hat{\mu}_{median} = 4.29$
$\hat{\mu}_{median} = 1.39$
AL (n = 16)
L (n = 9)
N (n = 8)
Group
Pairwise test: **Dunn**, Bars shown: **significant**
S3
$\chi^2_{Kruskal-Wallis}(2) = 0.79$, $p = 0.67$, $\hat{\epsilon}^2_{ordinal} = 0.02$, $CI_{99\%}$ [6.06e−03, 1.00], $n_{obs} = 35$
Number of copies
$\hat{\mu}_{median} = 4.27$
$\hat{\mu}_{median} = 4.28$
$\hat{\mu}_{median} = 0.00$
AL (n = 16)
L (n = 10)
N (n = 9)
Group
Pairwise test: **Dunn**, Bars shown: **significant**

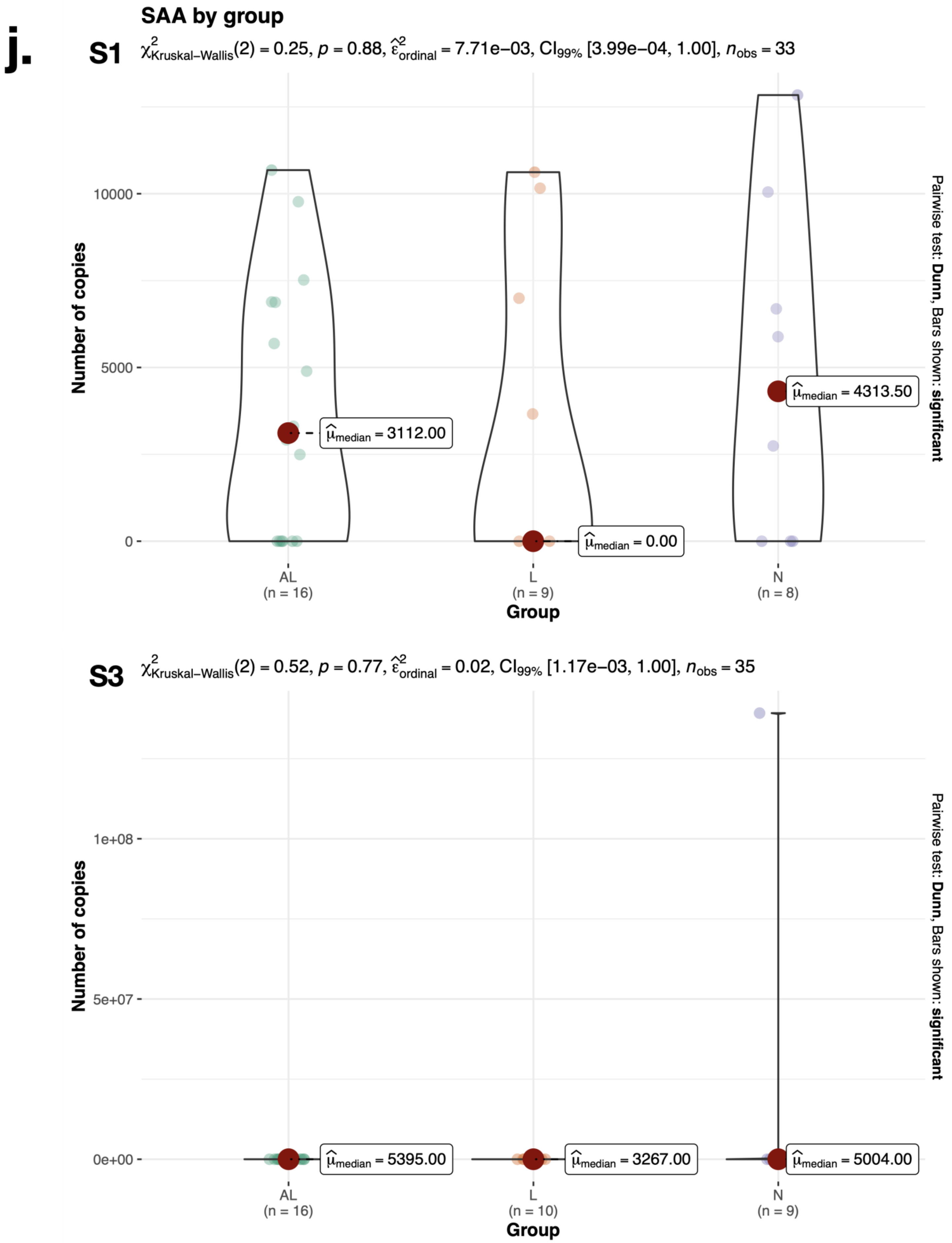
j.
SAA by group
S1
$\chi^2_{\text{Kruskal-Wallis}}(2) = 0.25$, $p = 0.88$, $\widehat{\varepsilon}^2_{\text{ordinal}} = 7.71\text{e}{-03}$, $\text{CI}_{99\%}$ [3.99e−04, 1.00], $n_{\text{obs}} = 33$
Number of copies
10000
5000
0
$\widehat{\mu}_{\text{median}} = 3112.00$
$\widehat{\mu}_{\text{median}} = 0.00$
$\widehat{\mu}_{\text{median}} = 4313.50$
AL
(n = 16)
L
(n = 9)
N
(n = 8)
Group
Pairwise test: **Dunn**, Bars shown: **significant**
S3
$\chi^2_{\text{Kruskal-Wallis}}(2) = 0.52$, $p = 0.77$, $\widehat{\varepsilon}^2_{\text{ordinal}} = 0.02$, $\text{CI}_{99\%}$ [1.17e−03, 1.00], $n_{\text{obs}} = 35$
Number of copies
1e+08
5e+07
0e+00
$\widehat{\mu}_{\text{median}} = 5395.00$
$\widehat{\mu}_{\text{median}} = 3267.00$
$\widehat{\mu}_{\text{median}} = 5004.00$
AL
(n = 16)
L
(n = 10)
N
(n = 9)
Group
Pairwise test: **Dunn**, Bars shown: **significant**
