# Supplementary material for: Study of the Genetic Expression of Antiretroviral Restriction Factors and Acute Phase Proteins in Cattle Infected with Bovine Leukemia Virus
Source: Pathogens. 2023 Mar 29;12(4):529. doi: 10.3390/pathogens12040529 (PMC10146972; doi:10.3390/pathogens12040529)
Supplement: Supplementary file 1 [file pathogens-12-00529-s001.zip › Table S1.pdf]

Table S1. Primers used in PCR techniques to amplify genetic regions of ARF, APP, BRDC virus, BLV proviral, and viral load.

ID	Virus	Sequence (5´- 3´)	Gene	Amplicon	Tm
FWA3	BRSV	GTACCAATGTCAACCAAATTCC	N	245 bp	59
RVA3		TTTGCACATCGTAATTGGGTAT			59
FWA2	BPIV-3	AAAGGAGTAGTTCAGATTCTAGATG	M	199 bp	59
FWA2		ATATAGAGCCAGTTGCGTT			59
FWA1	BoHV-1	GCCGTACCACTCCCACGTAT	gE	159 bp	59
RV A1		CACGTCTCGTATATGCGGATGA			59
FWA4	BVDV	GCTAGCCATGCCCTTAGTAGGACTAGC	5´NTR	293 bp	59
Rv A4		AACTCCATGTGCCATGTACAGCAGAG			59
FWA6	HEXIM-1	TCTCCCCTTAAGACCCAGGT	HEXIM-1	123 bp	55
RV A6		TTTCTTGCCCACCTGTCTCT			55
FWA7	APOBEC-Z1	CCACATAAGCCTGCACATCC	APOBEC-Z1	114 bp	55
RV A7		GTCATGATGGTGATTCGGGC			55
FWA8	APOBEC-Z2	AACTCCGCTTCCTGTCTTG	APOBEC-Z2	151 bp	55
RV A8		ATGCTCAGCGTCACATTCTG			55
FWA9	APOBEC-Z3	TTTAAGATGGGGCTGCAGGA	APOBEC-Z3	162 bp	55
RV A9		TGGGACAAGCTGGAGCAATA			55
FWA10	BST2	AGTTCCTGATGCCCATAGACA	BST2	195 bp	55
RV A10		CCTCCTGACACTCCTTCTGT			55
FWA11	HEXIM-2	GAGACTACCTGGATCTGGAGAG	HEXIM-2	113 bp	55
RV A11		CAGTTCCTCCACTTGGTAACAG			55
FWA12	SAA-2	GGGTGCAGACAAATACTTCC	SAA-2	134 bp	55
RV A12		CCTTAAACAGAGGGTCTGTGA			55
FWA13	HP	TCCCAGCATAACCTCATCTC	HP	95 bp	57
RV A13		TTGTCACTACTGTGACCCA			57
Fw1 env*	BLV	GGGTGCGAGAAACCATTCA	*env*	99 bp	62
Rv1 env *	BLV	GGCGAGAGGTCAAGCATTT			62
Fw2 env**	BLV	CCAAGGGCTCTGATAAACTC	*env*	745 bp	58
Rv2 env**	BLV	ATGATGTGACAGAGGGAACC			58
Fw-TBP	TBP	AGTCGACGTCCCAGCAG	*TBP*	112 bp	58
Rv-TBP	TBP	GAGGGATACAAGGGAGTGGT	*TBP*	112 bp	58
Fw-HPRT1	HPRT-1	CGAGATGTGATGAAGGAGATGG	HPRT-1	85 bp	58
Rv-HPRT1	HPRT-1	ACAGGTCGGCAAAGAACTTAT	HPRT-1	85 bp	58

ID: identification; Fw: forward; Rv: reverse; BRSV: bovine respiratory syncytial virus; BPIV-3: bovine parainfluence 3; BoHV-1: bovine herpesvirus type 1; BVDV: bovine diarrhea virus; BLV: bovine leukemia virus; bp: Base pairs. * primer FW position 6112-6130, Rv position 6192-6211; ** Fw position 5852–5871, Rv position 6506–6488, primer position was based on the OIE reference sequence with accession number K02120.
