# Supplementary material for: Study of the Genetic Expression of Antiretroviral Restriction Factors and Acute Phase Proteins in Cattle Infected with Bovine Leukemia Virus
Source: Pathogens. 2023 Mar 29;12(4):529. doi: 10.3390/pathogens12040529 (PMC10146972; doi:10.3390/pathogens12040529)
Supplement: Supplementary file 1 [file pathogens-12-00529-s001.zip › Table S2.pdf]

Table S2. Results of the shapiro-wilk test to determine normality, and the Bartlett test to determine the homoscedasticity of the samples.

		**shapiro-wilk**		**Bartlett**	
		W	p-value	K-squire	p-value
**Number of copies**	**Number of lymphocytes**	0.86718	3.233e-06	21.739	1.903 e-05
	**Provirus**	0.44121	1.175e-14	Inf	< 2.2e-16
	**Virus**	0.45532	1.805e-14	Inf	< 2.2e-16
	**APOBEC-Z1**	0.10377	< 2.2e-16	433.36	< 2.2e-16
	**APOBEC-Z2**	0.1049	< 2.2e-16	Inf	< 2.2e-16
	**APOBEC-Z3**	0.11865	< 2.2e-16	206.81	< 2.2e-16
	**BST2**	0.15117	< 2.2e-16	122.59	< 2.2e-16
	**HEXIM-1**	0.11591	< 2.2e-16	184.74	< 2.2e-16
	**HEXIM-2**	0.11549	< 2.2e-16	217.05	< 2.2e-16
	**SAA**	0.10111	< 2.2e-16	576.62	< 2.2e-16
	**HP**	0.10028	< 2.2e-16	Inf	< 2.2e-16

W= Shapiro-Wilk normality test value

K-square= value of Bartlett's test for homogeneity of variances
