# Supplementary material for: Study of the Genetic Expression of Antiretroviral Restriction Factors and Acute Phase Proteins in Cattle Infected with Bovine Leukemia Virus
Source: Pathogens. 2023 Mar 29;12(4):529. doi: 10.3390/pathogens12040529 (PMC10146972; doi:10.3390/pathogens12040529)
Supplement: Supplementary file 1 [file pathogens-12-00529-s001.zip › Table S3.pdf]

Table S3a: *Means, standard deviations, and correlations with confidence intervals: sampling 1 negative group.*

Variable	*M*	*SD*	1	2	3	4	5	6	7	8	9	10
1. Lymphocyte	3164.78	1621.57										
2. Proviral	0.00	0.00	NA<br>[NA, NA]									
3. Viral	0.00	0.00	NA<br>[NA, NA]	NA<br>[NA, NA]								
4. Z1	77307.50	22178.79	.49<br>[-.33, .89]	NA<br>[NA, NA]	NA<br>[NA, NA]							
5. Z2	0.00	0.00	NA<br>[NA, NA]	NA<br>[NA, NA]	NA<br>[NA, NA]	NA<br>[NA, NA]						
6. Z3	77.37	162.76	-.16<br>[-.78, .61]	NA<br>[NA, NA]	NA<br>[NA, NA]	.00<br>[-.70, .71]	NA<br>[NA, NA]					
7. BST2	113.86	104.87	.33<br>[-.49, .84]	NA<br>[NA, NA]	NA<br>[NA, NA]	.76*<br>[.13, .95]	NA<br>[NA, NA]	.00<br>[-.70, .71]				
8. HEXIM1	1.11	1.90	-.76*<br>[-.95, -.13]	NA<br>[NA, NA]	NA<br>[NA, NA]	-.36<br>[-.85, .46]	NA<br>[NA, NA]	-.11<br>[-.76, .65]	.06<br>[-.67, .73]			
9. HEXIM2	2.42	2.81	.10<br>[-.65, .75]	NA<br>[NA, NA]	NA<br>[NA, NA]	.08<br>[-.66, .74]	NA<br>[NA, NA]	.05<br>[-.68, .73]	.47<br>[-.35, .88]	.45<br>[-.37, .88]		
10. HP	0.00	0.00	NA<br>[NA, NA]	NA<br>[NA, NA]	NA<br>[NA, NA]	NA<br>[NA, NA]	NA<br>[NA, NA]	NA<br>[NA, NA]	NA<br>[NA, NA]	NA<br>[NA, NA]	NA<br>[NA, NA]	
11. SAA	4775.50	4932.38	.07<br>[-.67, .74]	NA<br>[NA, NA]	NA<br>[NA, NA]	.65<br>[-.10, .93]	NA<br>[NA, NA]	-.47<br>[-.88, .35]	.37<br>[-.45, .85]	.00<br>[-.70, .71]	.03<br>[-.69, .72]	NA<br>[NA, NA]

Note. M and SD are used to represent mean and standard deviation, respectively. Values in square brackets indicate the 95% confidence interval for each correlation. The confidence interval is a plausible range of population correlations that could have caused the sample correlation (Cumming, 2014).
* indicates $p < .05$. ** indicates $p < .01$. NA: not available.

Table S3b: *Means, standard deviations, and correlations with confidence intervals: sampling 1 aleukemic group (AL).*

Variable	*M*	*SD*	1	2	3	4	5	6	7	8	9	10
1. Lymphocyte	4905.39	3158.01										
2. Proviral	616389.01	1136645.1<br>9	.06<br>[-.45, .54]									
3. Viral	837.25	1653.54	.04<br>[-.47, .52]	.90**<br>[.73, .97]								
4. Z1	53292.50	16284.02	.04<br>[-.46, .53]	.47<br>[-.03, .78]	.30<br>[-.23, .69]							
5. Z2	4.70	13.24	-.21<br>[-.64, .32]	-.08<br>[-.55, .43]	-.19<br>[-.63, .34]	.45<br>[-.06, .77]						
6. Z3	222.03	298.13	.28<br>[-.25, .68]	-.12<br>[-.58, .40]	-.20<br>[-.63, .33]	.08<br>[-.43, .56]	.36<br>[-.16, .73]					
7. BST2	76.79	95.73	.36<br>[-.16, .73]	-.24<br>[-.66, .29]	-.21<br>[-.64, .32]	-.12<br>[-.58, .40]	-.02<br>[-.51, .48]	.47<br>[-.03, .79]				
8. HEXIM1	0.68	1.44	.16<br>[-.37, .61]	-.23<br>[-.65, .30]	-.22<br>[-.65, .31]	.02<br>[-.48, .51]	.27<br>[-.26, .67]	.56*<br>[.09, .83]	.17<br>[-.36, .61]			
9. HEXIM2	4.45	3.31	.10<br>[-.42, .57]	-.22<br>[-.65, .31]	.03<br>[-.47, .52]	-.37<br>[-.73, .15]	.02<br>[-.48, .51]	-.18<br>[-.62, .35]	-.04<br>[-.53, .46]	.41<br>[-.11, .75]		
10. HP	0.00	0.00	NA<br>[NA, NA]	NA<br>[NA, NA]	NA<br>[NA, NA]	NA<br>[NA, NA]	NA<br>[NA, NA]	NA<br>[NA, NA]	NA<br>[NA, NA]	NA<br>[NA, NA]	NA<br>[NA, NA]	
11. SAA	3814.44	3740.99	-.02<br>[-.51, .48]	-.25<br>[-.66, .28]	-.32<br>[-.70, .21]	-.36<br>[-.72, .17]	.09<br>[-.43, .56]	.12<br>[-.40, .58]	.24<br>[-.29, .66]	-.15<br>[-.60, .38]	.04<br>[-.47, .52]	NA<br>[NA, NA]

Note. M and SD are used to represent mean and standard deviation, respectively. Values in square brackets indicate the 95% confidence interval for each correlation. The confidence interval is a plausible range of population correlations that could have caused the sample correlation (Cumming, 2014).
* indicates $p < .05$. ** indicates $p < .01$. NA: not available.

Table S3c: *Means, standard deviations, and correlations with confidence intervals: sampling 1 group persistent lymphocytosis (PL).*

Variable	*M*	*SD*	1	2	3	4	5	6	7	8	9	10
1. Lymphocyte	16535.00	5154.02										
2. Proviral	2378893.33	2578246.71	-.14<br>[-.74, .58]									
3. Viral	5045.11	7205.86	-.58<br>[-.90, .14]	.41<br>[-.35, .85]								
4. Z1	43920.78	22255.57	-.57<br>[-.89, .16]	-.22<br>[-.77, .52]	.06<br>[-.63, .70]							
5. Z2	0.00	0.00	NA<br>[NA, NA]	NA<br>[NA, NA]	NA<br>[NA, NA]	NA<br>[NA, NA]						
6. Z3	350.10	561.67	-.27<br>[-.79, .48]	-.56<br>[-.89, .17]	-.23<br>[-.78, .51]	.58<br>[-.13, .90]	NA<br>[NA, NA]					
7. BST2	181.92	204.37	-.09<br>[-.71, .61]	-.08<br>[-.71, .62]	.66<br>[.00, .92]	.17<br>[-.55, .75]	NA<br>[NA, NA]	-.03<br>[-.68, .64]				
8. HEXIM1	0.46	0.98	.21<br>[-.53, .77]	-.46<br>[-.86, .29]	-.20<br>[-.76, .54]	.09<br>[-.61, .71]	NA<br>[NA, NA]	.15<br>[-.57, .74]	.04<br>[-.64, .68]			
9. HEXIM2	3.89	3.21	-.36<br>[-.82, .40]	.60<br>[-.11, .90]	.68*<br>[.04, .93]	.03<br>[-.65, .68]	NA<br>[NA, NA]	-.31<br>[-.81, .44]	.34<br>[-.42, .82]	-.61<br>[-.91, .09]		
10. HP	0.00	0.00	NA<br>[NA, NA]	NA<br>[NA, NA]	NA<br>[NA, NA]	NA<br>[NA, NA]	NA<br>[NA, NA]	NA<br>[NA, NA]	NA<br>[NA, NA]	NA<br>[NA, NA]	NA<br>[NA, NA]	
11. SAA	3492.78	4589.76	-.07<br>[-.70, .62]	.19<br>[-.54, .76]	.09<br>[-.61, .71]	.12<br>[-.59, .73]	NA<br>[NA, NA]	-.44<br>[-.85, .32]	.24<br>[-.51, .78]	-.39<br>[-.84, .37]	.16<br>[-.56, .75]	NA<br>[NA, NA]

Note. M and SD are used to represent mean and standard deviation, respectively. Values in square brackets indicate the 95% confidence interval for each correlation. The confidence interval is a plausible range of population correlations that could have caused the sample correlation (Cumming, 2014).
* indicates $p < .05$. ** indicates $p < .01$. NA: not available.

Table S3d: *Means, standard deviations, and correlations with confidence intervals: sampling 3 negative group.*

Variable	*M*	*SD*	1	2	3	4	5	6	7	8	9	10
1. Lymphocyte	5359.67	1223.49										
2. Proviral	0.00	0.00	NA<br>[NA, NA]									
3. Viral	0.00	0.00	NA<br>[NA, NA]	NA<br>[NA, NA]								
4. Z1	6504834.44	19381955.65	.04<br>[-.64, .69]	NA<br>[NA, NA]	NA<br>[NA, NA]							
5. Z2	0.00	0.00	NA<br>[NA, NA]	NA<br>[NA, NA]	NA<br>[NA, NA]	NA<br>[NA, NA]						
6. Z3	7.64	22.92	.77*<br>[.23, .95]	NA<br>[NA, NA]	NA<br>[NA, NA]	-.12<br>[-.73, .59]	NA<br>[NA, NA]					
7. BST2	167.41	250.72	.47<br>[-.28, .86]	NA<br>[NA, NA]	NA<br>[NA, NA]	-.25<br>[-.78, .50]	NA<br>[NA, NA]	.31<br>[-.45, .81]				
8. HEXIM1	0.55	1.62	.16<br>[-.56, .74]	NA<br>[NA, NA]	NA<br>[NA, NA]	-.11<br>[-.72, .59]	NA<br>[NA, NA]	-.13<br>[-.73, .59]	.83**<br>[.38, .96]			
9. HEXIM2	3.14	3.73	.47<br>[-.28, .86]	NA<br>[NA, NA]	NA<br>[NA, NA]	-.32<br>[-.81, .44]	NA<br>[NA, NA]	.40<br>[-.36, .84]	-.12<br>[-.73, .59]	-.32<br>[-.81, .43]		
10. HP	0.00	0.00	NA<br>[NA, NA]	NA<br>[NA, NA]	NA<br>[NA, NA]	NA<br>[NA, NA]	NA<br>[NA, NA]	NA<br>[NA, NA]	NA<br>[NA, NA]	NA<br>[NA, NA]	NA<br>[NA, NA]	
11. SAA	15472451.06	46397831.76	.04<br>[-.64, .69]	NA<br>[NA, NA]	NA<br>[NA, NA]	1.00**<br>[1.00, 1.00]	NA<br>[NA, NA]	-.13<br>[-.73, .59]	-.25<br>[-.78, .50]	-.11<br>[-.72, .59]	-.32<br>[-.81, .44]	NA<br>[NA, NA]

Note. M and SD are used to represent mean and standard deviation, respectively. Values in square brackets indicate the 95% confidence interval for each correlation. The confidence interval is a plausible range of population correlations that could have caused the sample correlation (Cumming, 2014).
* indicates $p < .05$. ** indicates $p < .01$. NA: not available.

Table S3e. *Means, standard deviations, and correlations with confidence intervals: sampling 3 aleukemic group (AL).*

Variable	*M*	*SD*	1	2	3	4	5	6	7	8	9	10
1. Lymphocyte	7566.19	2596.64										
2. Proviral	402277.00	696725.51	.48<br>[-.02, .79]									
3. Viral	8123.06	14127.92	.41<br>[-.10, .76]	.96**<br>[.90, .99]								
4. Z1	91033.12	153826.40	.24<br>[-.29, .66]	.09<br>[-.43, .56]	.01<br>[-.49, .50]							
5. Z2	362.88	1451.50	.22<br>[-.31, .65]	.08<br>[-.43, .55]	-.02<br>[-.51, .48]	.99**<br>[.98, 1.00]						
6. Z3	6197.56	24683.37	.22<br>[-.31, .65]	.08<br>[-.43, .56]	-.02<br>[-.51, .48]	.99**<br>[.98, 1.00]	1.00**<br>[1.00, 1.00]					
7. BST2	874.32	3155.61	.23<br>[-.30, .65]	.09<br>[-.43, .56]	-.01<br>[-.50, .49]	1.00**<br>[.99, 1.00]	1.00**<br>[1.00, 1.00]	1.00**<br>[1.00, 1.00]				
8. HEXIM1	20.53	81.35	.22<br>[-.31, .65]	.08<br>[-.43, .55]	-.02<br>[-.51, .48]	.99**<br>[.98, 1.00]	1.00**<br>[1.00, 1.00]	1.00**<br>[1.00, 1.00]	1.00**<br>[1.00, 1.00]			
9. HEXIM2	63.66	239.35	.23<br>[-.30, .65]	.09<br>[-.43, .56]	-.01<br>[-.50, .49]	.99**<br>[.98, 1.00]	1.00**<br>[1.00, 1.00]	1.00**<br>[1.00, 1.00]	1.00**<br>[1.00, 1.00]	1.00**<br>[1.00, 1.00]		
10. HP[&]	1744.38	6977.50	.15<br>[-.37, .60]	.83**<br>[.57, .94]	.82**<br>[.56, .94]	-.06<br>[-.54, .45]	-.07<br>[-.54, .44]	-.07<br>[-.54, .44]	-.05<br>[-.53, .45]	-.07<br>[-.54, .44]	-.06<br>[-.54, .45]	
11. SAA	42266.75	149198.29	.23<br>[-.30, .65]	.08<br>[-.43, .55]	-.02<br>[-.51, .48]	.99**<br>[.98, 1.00]	1.00**<br>[1.00, 1.00]	1.00**<br>[1.00, 1.00]	1.00**<br>[1.00, 1.00]	1.00**<br>[1.00, 1.00]	1.00**<br>[1.00, 1.00]	-.08<br>[-.55, .44]

Note. M and SD are used to represent mean and standard deviation, respectively. Values in square brackets indicate the 95% confidence interval for each correlation. The confidence interval is a plausible range of population correlations that could have caused the sample correlation (Cumming, 2014).
* indicates $p < .05$. ** indicates $p < .01$. NA: not available. &: determination carried out in a single bovine.

Table S3f. *Means, standard deviations, and correlations with confidence intervals: sampling 3 group persistent lymphocytosis (PL).*

Variable	*M*	*SD*	1	2	3	4	5	6	7	8	9	10
1. Lymphocyte	18931.60	6338.98										
2. Proviral	2905680.00	5060288.29	.42<br>[-.29, .83]									
3. Viral	48980.50	47596.10	.63<br>[-.00, .90]	.76*<br>[.25, .94]								
4. Z1	43547.00	22684.39	-.04<br>[-.65, .61]	.10<br>[-.57, .68]	.24<br>[-.46, .75]							
5. Z2	10.42	32.95	-.27<br>[-.77, .44]	-.17<br>[-.72, .52]	-.25<br>[-.76, .45]	-.41<br>[-.83, .29]						
6. Z3	713.56	1478.42	.31<br>[-.40, .78]	-.09<br>[-.68, .57]	-.26<br>[-.77, .44]	-.58<br>[-.89, .07]	-.17<br>[-.72, .52]					
7. BST2	29.84	53.21	-.23<br>[-.75, .47]	-.25<br>[-.76, .45]	-.29<br>[-.78, .42]	-.41<br>[-.83, .29]	.62<br>[-.02, .90]	-.30<br>[-.78, .41]				
8. HEXIM1	1.14	2.32	-.15<br>[-.71, .53]	-.23<br>[-.75, .47]	-.31<br>[-.79, .39]	-.48<br>[-.85, .21]	.96**<br>[.85, .99]	.04<br>[-.60, .66]	.53<br>[-.15, .87]			
9. HEXIM2	3.48	3.24	-.04<br>[-.65, .61]	.04<br>[-.60, .65]	-.00<br>[-.63, .63]	-.39<br>[-.82, .32]	.09<br>[-.57, .68]	.29<br>[-.42, .78]	.27<br>[-.43, .77]	.06<br>[-.59, .67]		
10. HP	0.00	0.00	NA<br>[NA, NA]	NA<br>[NA, NA]	NA<br>[NA, NA]	NA<br>[NA, NA]	NA<br>[NA, NA]	NA<br>[NA, NA]	NA<br>[NA, NA]	NA<br>[NA, NA]	NA<br>[NA, NA]	
11. SAA	4232.30	4169.27	-.39<br>[-.82, .31]	.12<br>[-.55, .69]	-.06<br>[-.67, .59]	-.32<br>[-.79, .38]	.78**<br>[.28, .94]	-.20<br>[-.74, .49]	.27<br>[-.43, .77]	.67*<br>[.06, .91]	.16<br>[-.52, .72]	NA<br>[NA, NA]

Note. M and SD are used to represent mean and standard deviation, respectively. Values in square brackets indicate the 95% confidence interval for each correlation. The confidence interval is a plausible range of population correlations that could have caused the sample correlation (Cumming, 2014).
* indicates $p < .05$. ** indicates $p < .01$. NA: not available.
