# Supplementary material for: Study of the Genetic Expression of Antiretroviral Restriction Factors and Acute Phase Proteins in Cattle Infected with Bovine Leukemia Virus
Source: Pathogens. 2023 Mar 29;12(4):529. doi: 10.3390/pathogens12040529 (PMC10146972; doi:10.3390/pathogens12040529)
Supplement: Supplementary file 1 [file pathogens-12-00529-s001.zip › Table S4.pdf]

Table S4. Efficiency data of qPCR designed to determine the expression of antiretroviral restriction factors and acute phase proteins.

	Slope	Efficiency	Error	R^2	Y-Intercept
APOBEC-Z1	-3.3989	1.97	0.11	1.00	36.41
APOBEC-Z2	-3.3571	1.99	0.45	1.00	36.55
APOBEC-Z3	-3.8850	1.81	1.62	0.97	39.26
BST2	-3.8721	1.81	0.78	0.99	41.62
HEXIM-1	-3.9204	1.80	0.71	0.99	44.54
HEXIM-2	-3.4921	1.93	0.31	1.00	40.69
SAA	-3.7941	1.83	0.37	0.99	43.86
HP	-3.6320	1.89	0.81	0.98	56.14
