# Supplementary figures and images for: Study of the Genetic Expression of Antiretroviral Restriction Factors and Acute Phase Proteins in Cattle Infected with Bovine Leukemia Virus
Source: Pathogens. 2023 Mar 29;12(4):529. doi: 10.3390/pathogens12040529 (PMC10146972; doi:10.3390/pathogens12040529)

Figure S1

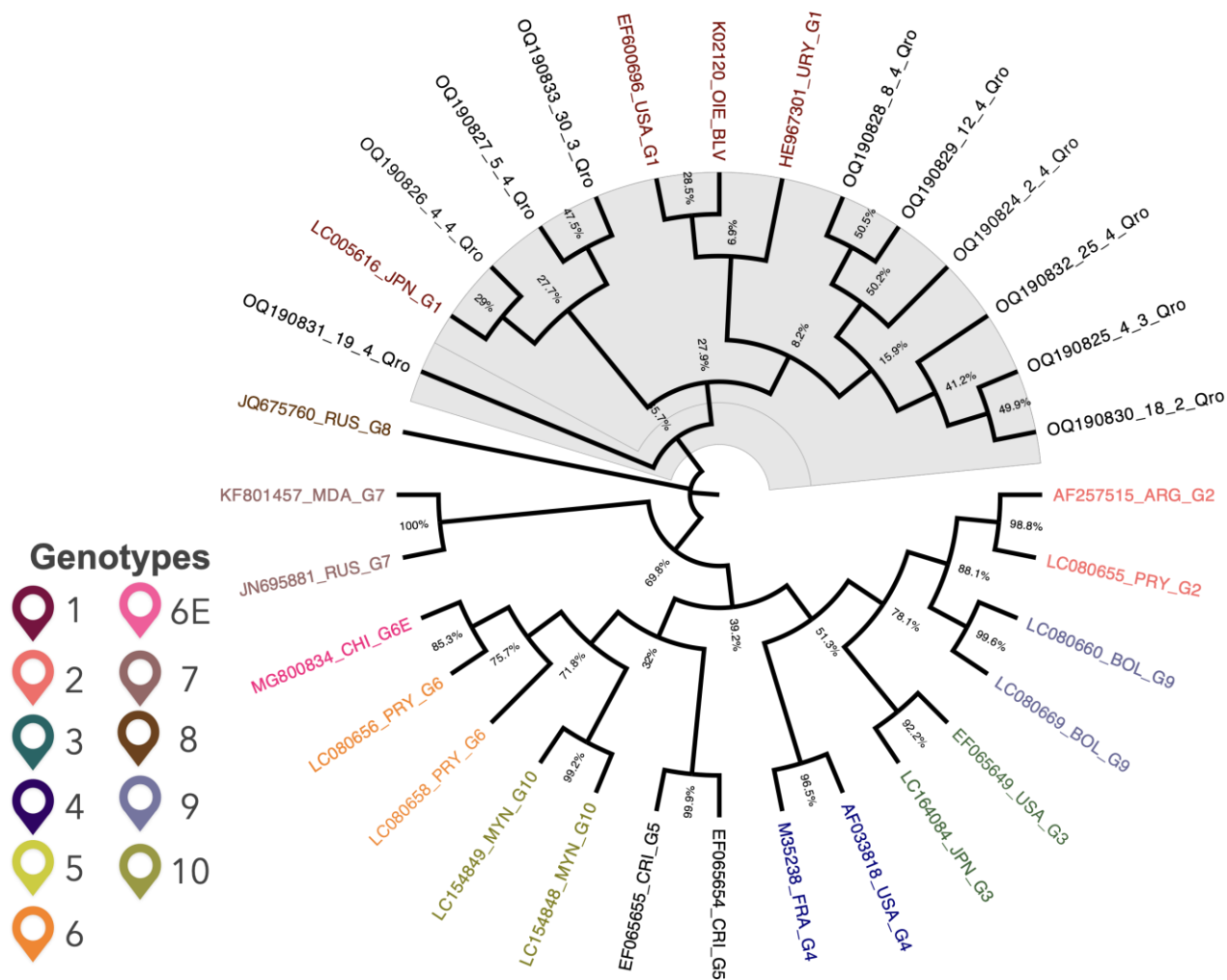

Supplement: Supplementary file 1 [file pathogens-12-00529-s001.zip › Figure S1.pdf]
